# Mucosal immune responses induced by oral administration recombinant *Bacillus subtilis* expressing the COE antigen of PEDV in newborn piglets

**DOI:** 10.1042/BSR20182028

**Published:** 2019-03-15

**Authors:** Jialu Wang, Lulu Huang, Chunxiao Mou, En Zhang, Yongheng Wang, Yanan Cao, Qian Yang

**Affiliations:** MOE Joint International Research Laboratory of Animal Health and Food Safety, College of Veterinary Medicine, Nanjing Agricultural University, Weigang 1 Nanjing, Jiangsu 210095, P.R. China

**Keywords:** mucosal immunity, Porcine epidemic diarrhea virus, piglets, recombinant Bacillus subtilis, vaccines

## Abstract

Porcine epidemic diarrhea (PED) is a highly contagious disease in newborn piglets and causes substantial economic losses in the world. PED virus (PEDV) spreads by fecal–oral contact and can be prevented by oral immunization. Therefore, it is necessary to develop an effective oral vaccine against PEDV infection. Currently, *Bacillus subtilis* as recombinant vaccine carrier has been used for antigen delivery and proved well in immune effect and safety. The present study evaluated the immunogenicity of recombinant *Bacillus subtilis (B. subtilis*-RC) in piglets via oral administration. After oral immunization in piglets, *B. subtilis*-RC significantly increased the local mucosal immune responses. Oral administration with *B. subtilis*-RC significantly improved the level of specific mucosal immunoglobulin A (IgA) antibodies against PEDV infection, through enlarging the area of Peyer’s patches (PPs) and increasing the number of ileum IgA^+^ secreting (SIgA) cells. In the meantime, *B. subtilis*-RC remarkably increased the number of intraepithelial lymphocytes (IELs). We also observed that oral administration of *B. subtilis*-RC significantly increased CD3^+^T lymphocytes’ numbers and up-regulated the ratio of CD4^+^/CD8^+^ T cells. Furthermore, high titers of specific serum immunoglobulin G (IgG) revealed satisfactory systemic immune response against PEDV infection. In summary, our study demonstrated that oral administration of *B. subtilis*-RC could trigger a high level of local and systemic immune responses and would be a promising candidate vaccine against PEDV infection in piglets.

## Introduction

Porcine epidemic diarrhea (PED) characterized by highly fatal acute diarrhea in piglets, results in enormous losses in the worldwide pig industry [1]. The causative agent PED virus (PEDV) belongs to the porcine coronaviruses (CoVs). PEDV infection mainly spreads through the digestive tract [2], and damages the host intestine mucosal surfaces by infecting the intestine epithelial cells [3]. Therfore enhancing intestinal mucosal immunity can elicit effective mucosal immune responses against PEDV infection [4]. Currently, traditional vaccines (intramuscular route or subcutaneous injection) have been developed and applied widely in the market [5]. These vaccines administered parenterally cannot effectively induce high titers of maternal antibodies and virus-specific IgA antibodies, resulting in inadequate mucosal protection to against PEDV infection [6]. Furthermore, these maternal antibodies in the milk were always degraded by gastric acid and pepsin before entering the intestinal tract. Effective PEDV vaccines must provide adequate mucosal protection in the intestinal tract. However, the effective vaccines are currently lacking [7].

As a superior way of mucosal immunization, oral administration can protect the gut and stimulate the common mucosal immune system [8]. Besides, oral immunization has several attractive features which include safety, and a straightforward, inexpensive, and needle-free approach [9]. Therefore, oral immunization often delivers large amounts of antigens to prevent the diarrheal diseases [10]. Nevertheless, there are several challenges by oral immunization, which consist of physical, chemical, and biological barriers when delivering antigens to the gastrointestinal (GI) tract (such as gastric acids, pepsin, and trypsin in the GI tract) [11].

It is a substantial problem that digestive acids and proteases can degrade antigen proteins for nutrient absorption [12]. Therefore, the vaccine delivery system has been applied to solve the problem. The system can protect antigens from the severe environment of the GI tract and deliver antigens to intestinal mucosa [13]. Currently, *Bacillus subtilis* (*B. subtilis*) is widely used as a vaccine delivery system for its unique characteristics.

As a nonpathogenic Gram-positive bacterium, *B. subtilis* has been regarded as a novel probiotic and food additive in humans and animals [14]. The *B. subtilis* has adjuvant activity and can deliver heterologous antigens to the GI tract, providing additional immunity stimulation [15]. Besides, research had shown that orally administered *B. subtilis* could also enhance immune regulation and gut health in pigs [16]. Moreover, oral administration of *B. subtilis* could elicit humoral and cellular immune responses to the maintenance of gut homeostasis by dendritic cells (DCs) [17]. DCs are the most important professional antigen-presenting cells and can effectively regulate antibody titers [18]. DCs naturally exist in the gut-associated lymphoid tissue (GALT), including Peyer’s patches (PPs), isolated lymphoid follicles (ILFs), mesenteric lymph nodes (MLNs), and scatter throughout the subepithelial lamina propria (LP) of the small intestine and colon [19]. Furthermore, *B. subtilis* is convenient for genetic manipulation and has developed a large variety of genetic tools [20]. Therefore, *B. subtilis* is widely used as an effective vaccine delivery system to induce mucosal immune responses and shows unique effect on the immune system.

In the present report, we explored the immune effect of a recombinant *B. subtilis* (*B. subtilis*-RC) which had been successfully constructed with expressing PEDV COE protein in piglets. Our research indicated that *B. subtilis*-RC was beneficial to the mucosal immune system development, and could effectively generate specific antibodies against PEDV infection, suggesting a potential approach for preventing PEDV infection.

## Materials and methods

### Virus, bacterium, and cell lines

The *B. subtilis* WB800 was kindly provided by Dr. Xuewen Gao (from the department of plant pathology, Nanjing Agricultural University) [21]. *B. subtilis*-RC previously constructed in our laboratory was able to express the gene *COE* (499–638 amino acids in S protein). Prior to oral administration, the recombinant strain was grown in LB broth at 37°C for 12 h, and then washed twice with PBS, and suspended in PBS to reach a final concentration of 1 × 10^10^ CFU/ml. The PEDV Zhejiang08 strain was provided by the Veterinary Medicine Research Centre of the Beijing Dabeinong Technology Group Co., Ltd. [22]. The virus was cultured in African green monkey kidney cells (Vero cells) and purified by using a discontinuous sucrose density gradient. The virus was UV-inactivated at UV dose of 4 J/cm^2^ for 24 h to achieve a complete loss of infectivity [23]. The purified virus concentration was measured using the BCA protein assay kit (Thermo Fisher, MA, U.S.A.).

### Reagents

FACS: 647 Mouse anti-Pig CD3ε (BB23-8E6-8C8), FITC Mouse anti-Pig CD4a (74-12-4), PE Mouse Anti-Pig CD8a (76*-*2-11) were purchased from BD. IHC: Rabbit anti-pig CD3 (SP7) mAbs were purchased from Abcam, Hong Kong. FITC Rabbit anti-pig CD4a were purchased from Santa. Mouse anti-Pig CD8α Antibody (76-2-11) were purchased from Novus.

ELISA: Rabbit anti-pig IgG (horseradish peroxidase (HRP)), Goat Anti-Pig IgA (HRP) were purchased from Abcam. Second antibody: DyLight 649–conjugated goat anti-mouse IgG antibody, DyLight 488–conjugated goat anti-rabbit IgG antibody, DyLight 594–conjugated goat anti-rabbit IgG antibody were purchased from Multi-science, Hangzhou, China. ABC-based system (biotinylated goat anti-rabbit IgG antibody) was used as the secondary antibody with DAB as a chromogen was purchased from Boster, Wuhan, China.

### Animals and vaccination programs

**Figure 1:**
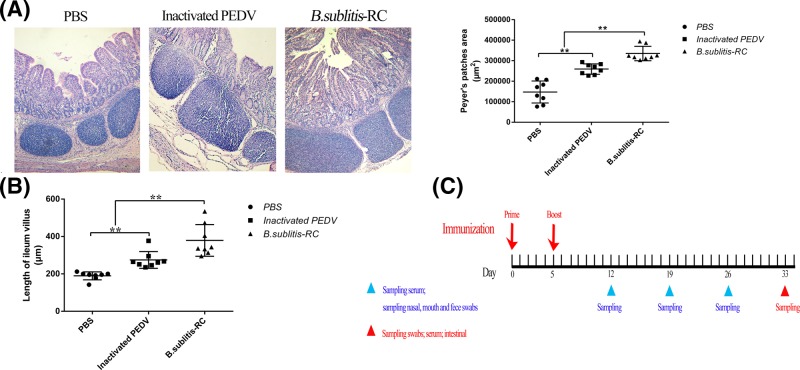
Oral administration *B. subtilis*-RC significantly promoted piglets intestinal development and schematic diagram of the immunization (**A**,**B**) The area of PPs and the length of ileum villus were counted from eight discontinuous HE staining slices which were selected from each group. (**C**) Schematic of the immunization, the red arrows indicated the time points of primary immunization and booster immunization, the blue triangle (under the line) indicated the time point of sampling three swabs (including nasal, mouth, and feces swabs) and serum, the red triangle (under the line) represent killing the piglets and sampling the three swabs, serum and intestinal. Data were shown as the mean ± S.D. Two individual experiments were performed, four piglets were used in each group for each individual protocol and each animal was analyzed individually. *P*-values: **P<0.01.

Specific pathogen-free (SPF) DLY piglets (Duroc and Landrace and Yorkshire) were kindly provided by Jiangsu Academy of Agricultural Sciences (Nanjing, China). The animal experiments had been approved by the Institutional Animal Care and Use Committee of Nanjing Agricultural University and followed the National Institutes of Health’s guidelines for the performance of animal experiments. Twelve newborn piglets were randomly divided into three groups (four piglets in each group), and housed under similar conditions in different stables in order to avoid probiotic cross-contamination. The piglets were orally dosed with 100 μl of *B. subtilis*-RC. The control groups of piglets were orally administered with inactivated PEDV (100 μg/dose) and equal volume of PBS. The immunization protocol was performed on the piglets that were 5 days old (Figure 1C), and signed as 0 day. Then booster immunizations were administered on 5 days.

Specimen collection was then performed every 7 days post boost immunization (Figure 1C). Blood samples were collected weekly from all piglets after the boost immunization and allowed to clot overnight at room temperature to collect serum. Blood samples were separated by centrifugation and stored at −20°C in order to detect the levels of specific IgG and IgA. Three swabs were collected every week lasting for 1 month, including nasal, oral, and feces swabs for the ELISA. The piglets were sacrificed in 33 days. The same location of the small intestine and ileum tissues from each piglet were fixed with Bonn’s liquid and 4% paraformaldehyde.

### Hematoxylin–eosin staining assay and intraepithelial lymphocytes number

The small intestine tissues in same location were fixed with Bouin Fixative Solution for 24 h, embedded in paraffin, and sectioned at 4-μm thickness. The sections were placed on glass slides. Hematoxylin–eosin staining was applied to the paraffin sections, then observing and taking photographs under optical microscope (OLYMPUS CX23). The number of intraepithelial lymphocytes (IELs) were counted in every 100 epithelial cells under the same multiple light microscope amongst ten pictures from each group [24].

### Immunohistochemistry

The immunohistochemistry detection was performed with the SABC kit (Boster Bioscience). Hydrogen peroxide was used to deactivate intrinsic peroxidase. Antigen retrieval was performed in a water bath using citrate-EDTA buffer (10 mM citric acid, 2 mM EDTA, 0.05% Tween 20, pH 6.2). Sections were incubated with diluted anti-IgA antibody (1:100; Abcam) overnight at 4°C. As negative controls, immunostaining performed by incubating samples with control antiserum instead of primary antibody. The addition of biotin-labeled secondary antibody to the slides was followed by adding HRP-labeled streptavidin. After staining with DAB, the slides were recorded using a digital camera (Leica-DM4000B) [25].

### Isolation of intestinal lymphocytes and detection by flow cytometry

The isolated intestines with PPs were transferred to ice-cold PBS. Then, remaining fat and connective tissue was removed and washed thoroughly with ice-cold PBS. Next, the intestine was cut longitudinally into 0.5-cm fragments. The fragments were incubated with 5 ml of 30 mM EDTA and placed in 5 ml digestion solution containing 4% FBS, 0.5 mg/ml each of Collagenase D (Roche) and DNase I (Sigma), and 50 U/ml Dispase (Fisher). The fragments were incubated with Dulbecco’s PBS (DPBS) for 20 min at 37°C by slow rotation (100 rpm). After incubating, the epithelial cells layer which contained the IELs were separated by intensive vortex and passed through a 70-μm cell strainer. Single cell suspension was collected and washed twice by DPBS, the solution was vortexed intensely and passed through a 40-μm cell strainer. Supernatants was washed by precooled RPMI medium 1640 (Thermo Fisher Scientific) and suspended by 10 ml of the 40% fraction of a 40:80 Percoll gradient, overlaid on 5 ml of the 80% fraction in a 15-ml Falcon tube. Percoll gradient separation was performed by centrifuging for 20 min at 2500 rpm. LP lymphocytes (LPLs) were collected at the interphase of the Percoll gradient, then washed and suspended in FACS buffer or T cell medium. In the meantime, flow cytometry analysis was performed on BD Facscalibur (BD Biosciences) instruments and analyzed by FlowJo software. All antibodies were purchased from BD Pharmingen or eBiosciences. Isolated single-cell suspensions were stained with anti-CD3-APC, anti-CD4-FITC, anti-CD8-PE, all at 1:100 dilution for 30 min on ice, and washed with PBS twice, and analyzed by FACS [26].

### Cytokines detection

Cytokines interleukin (IL) 10 (IL-10) and IL-1β (Abcam) were measured by ELISA according to the manufacturer’s instructions. Data were acquired on an automated ELISA plate reader at OD 450 nm immediately.

### Plaque reduction neutralization test

PEDV neutralizing antibodies were measured in intestine washing liquid by plaque reduction neutralization test (PRNT). The test was performed as previously described with minor modifications [27]. A total of 450 μl of intestine washing liquid was two-fold serially diluted and mixed with 50 μl viral suspension containing 10^3^ TCID_50_ PEDV virus for 1 h at 37°C in 12-well flat bottomed tissue culture plates. The mixture was then inoculated for 1 h at 37°C and 5% CO_2_. Then, the mixture was inoculated with Vero cells suspension (approximately 1.0 × 10^6^ ml^−1^) for another 3–4 days. After staining with Crystal Violet, the plates were observed under a microscope for cytopathic effect.

### Statistical analyses

Data were obtained as the means ± S.E.M. of three replicates per test in a single experiment. GraphPad Prism V6.0 (San Diego, CA, U.S.A.) used to perform statistical analyses. Tukey’s multiple comparison tests and one-way ANOVA were used to analyze the significance of the difference between means. *P*-values less than 0.05 (*P*<0.05) were considered significant and *P*-values less than 0.01 (*P*<0.01) as highly significant.

## Results

### Oral administration of *B. subtilis*-RC significantly promoted intestinal development in piglets

 PPs are a concentrate of lymphoid tissue and the primary site for immunoglobulin A (IgA) production which is crucial to regulate the homeostatic balance of intestine [28]. The area of PPs is a key immunity indicator. Oral administration with *B. subtilis*-RC significantly (*P*<0.01) increased the area of PPs compared with two control groups as shown in Figure 1A. In addition, the villi length of ileum got longer by oral administration with *B. subtilis*-RC (*P*<0.01) than the other two groups (Figure 1B). These primarily confirmed that *B. subtilis*-RC was beneficial to maintain the structure of intestine.

**Figure 2:**
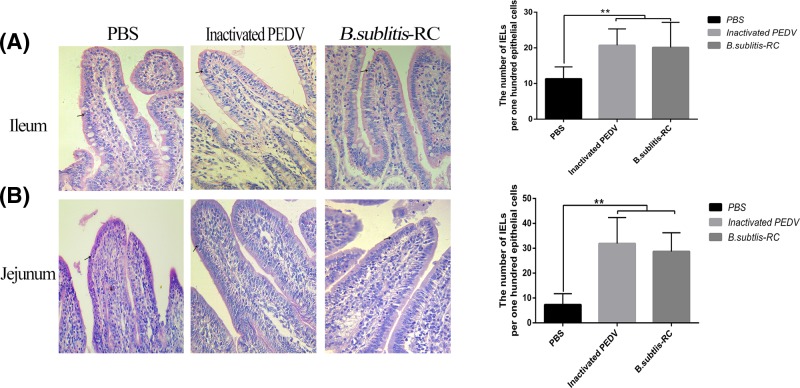
Oral administration *B. subtilis*-RC increased the number of IELs Each group chose eight discontinuous HE staining slices to count and averaged the numbers of IELs amongst 100 epithelial cells in ileum and jejunum. Data were shown as the mean ± S.D. *P*-value:**P<0.01.

Intestinal IELs are a large and diverse population of lymphoid cells residing within the intestinal epithelial cells (IECs), and forming the intestinal mucosal barrier [29]. IELs are important part of the gut mucosal immune system. No matter *B. subtilis*-RC or inactivated PEDV significantly (*P*<0.01) increased the number of ileum IELs in per 100 IECs (Figure 2). These results further proved the strength of immune enhancement by oral administration *B. subtilis*-RC.

### Oral immunization with *B. subtilis*-RC changed the local and systemic immune responses

**Figure 3:**
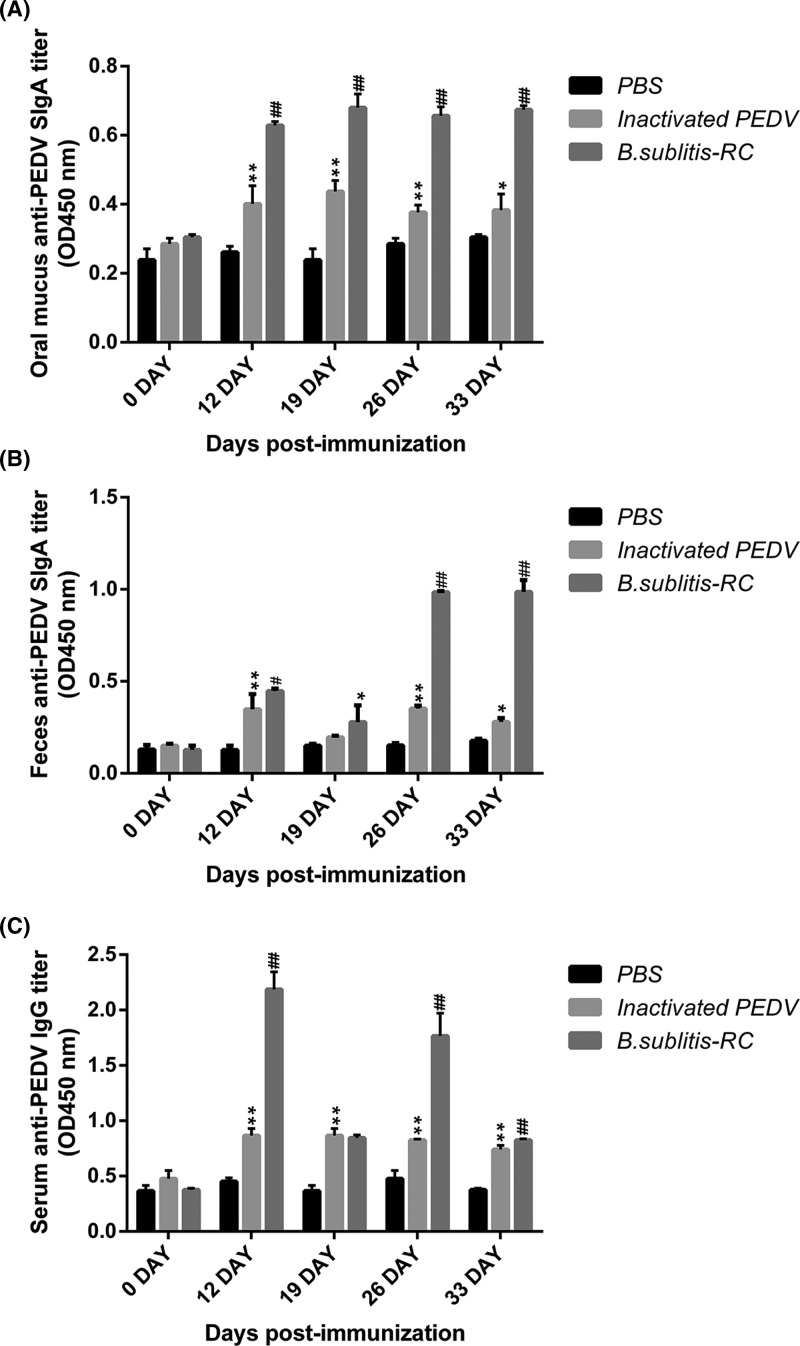
*B. subtilis*-RC changed the local and systemic immune responses after oral immunization (**A**,**B**) Determination of anti-PEDV-specific mucosal SIgA antibody in mouth (A) and feces (B) by ELISA using PEDV as the coating antigen. (**C**) Measurement of anti-PEDV IgG antibody in serum from immunized piglets by ELISA using PEDV as the coating antigen. Data were shown as the mean ± S.D. *P*-value: ^#^*P*<0.05, ^##^*P*<0.01 compared with inactivated PEDV; *P<0.05, **P<0.01 compared with PBS.

The level of specific anti-PEDV ileum IgA^+^ secreting (SIgA) antibody in piglets was measured by ELISA in the mouth and feces. As shown in Figure 3A,B, antigen-specific mucosal SIgA in the above sites was clearly higher than inactivated PEDV group (*P*<0.05 or *P*<0.01). As expected, the mouth had higher levels of SIgA than other sites.

After oral immunization, the level of serum anti-PEDV IgG antibody in piglets immunized with *B. subtilis-RC*, inactivated PEDV or PBS were determined by ELISA, as shown in Figure 3C. The results indicated that although the titers dropped during sampling period, the IgG level of *B. subtilis*-RC still significantly increased from 0 to 33 days than inactivated PEDV group (*P*<0.05 or *P*<0.01).

### Oral immunization with *B. subtilis*-RC increased SIgA cells and CD3^+^ T lymphocytes

**Figure 4:**
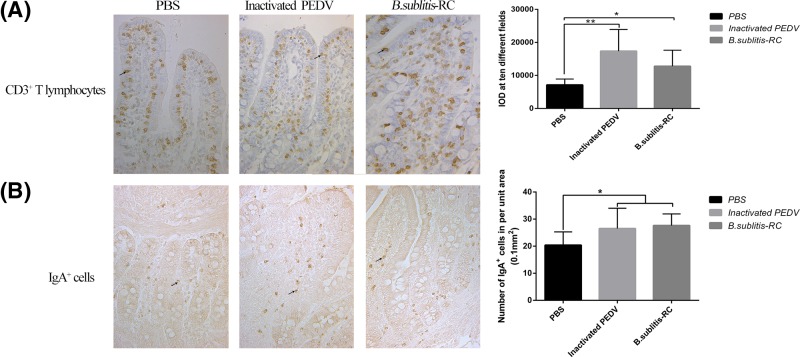
Oral administration *B. subtilis*-RC increased SIgA cells and CD3^+^ T lymphocytes Immunohistochemistry was used to detect SIgA cells and CD3^+^ T lymphocytes. The integrated optic density (IOD) of eight discontinuous slices were counted each group. (**A**) The CD3^+^ T lymphocytes in ileum. (**B**) The IgA^+^ cells in ileum. Data were shown as the mean ± S.D. *P*-value: *P<0.05, **P<0.01.

CD3^+^ T lymphocytes are the fundamental cell surface markers of T lymphocytes, therefore, the number of CD3^+^ T lymphocytes could represent the quantity of T lymphocytes. Consequently, we analyzed the number of CD3^+^ T lymphocytes in ileum. The data indicated that both *B. subtilis*-RC and inactivated PEDV could dramatically (*P*<0.05) increase CD3^+^ T lymphocytes compared with PBS group (Figure 4A). These changes showed confident evidence that oral administration with *B. subtilis*-RC had a good influence on intestinal mucosal immunity in piglets.

SIgA is the main immunoglobulin isotype in animals, largely secreted across the intestinal mucosal surface especially in the small intestine [30]. SIgA plays an important role in intestinal mucosal immunity and reflects on the intestinal mucosal immunity. After oral administration with *B. subtilis*-RC, the number of IgA secreting cells had quickly risen compared with the other two groups (*P*<0.05) (Figure 4B). These results showed that oral administration with *B. subtilis*-RC was conducive to intestinal mucosal immunity and could increase the number of IgA secreting cells to produce positive effects on against PEDV infection.

### Oral immunization with *B. subtilis*-RC stimulated differentiation of lymphocytes in the small intestinal mucosa

**Figure 5:**
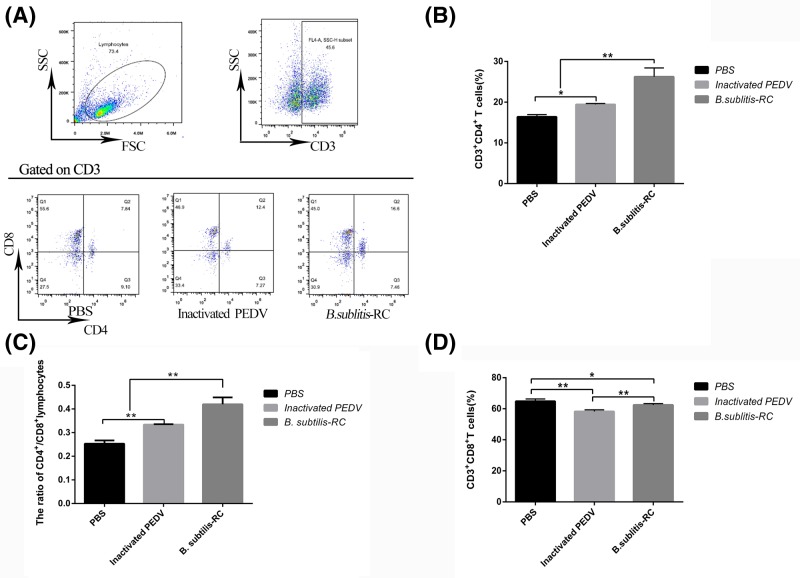
*B. subtilis*-RC stimulated differentiation of lymphocytes in the small intestinal mucosa via oral administration The lymphocyte of the ileum with PPs junction was isolated and the lymphocytes of CD3, CD4, and CD8 analyzed by three colors flow cytometry. (**A**) The lymphocytes of CD3, CD4, and CD8 analyzed by three colors flow cytometry. (**B**) The percentage of CD4^+^ in the CD3^+^ T lymphocytes. (**C**) The ratio of CD4^+^/CD8^+^ T lymphocytes. (**D**) The percentage of CD8^+^ in the CD3^+^ T lymphocytes. Data were shown as the mean ± S.D. *P*-value: *P<0.05, **P<0.01.

A great deal of immune cells are scattered in the epithelial cells. IECs indirectly or directly interact with innate and adaptive immune cells by presenting antigens to lymphocytes [31]. Consequently, learning about how the lymphocytes are distributed in the small intestinal mucosa is very meaningful for mucosal immunology. Previous data had shown that CD3^+^ T lymphocytes significantly (*P*<0.05) increased (Figure 4A), so we further analyzed the immunological classification of CD3^+^ T lymphocytes. The lymphocyte of the ileum with PPs junction was isolated and the lymphocytes of CD3, CD4, and CD8 were analyzed by three colors flow cytometry (Figure 5A). These results showed that CD3^+^CD4^+^ T cells have obviously (*P*<0.01) increased (Figure 5B), nevertheless the CD3^+^CD8^+^T cells remarkably (*P*<0.05) declined (Figure 5C). After calculation, the ratio of CD4^+^/CD8^+^T cells increased (Figure 5D). This ratio could also further measure the immunity levels of piglets.

### Oral immunization with *B. subtilis*-RC regulated cytokine responses

**Figure 6:**
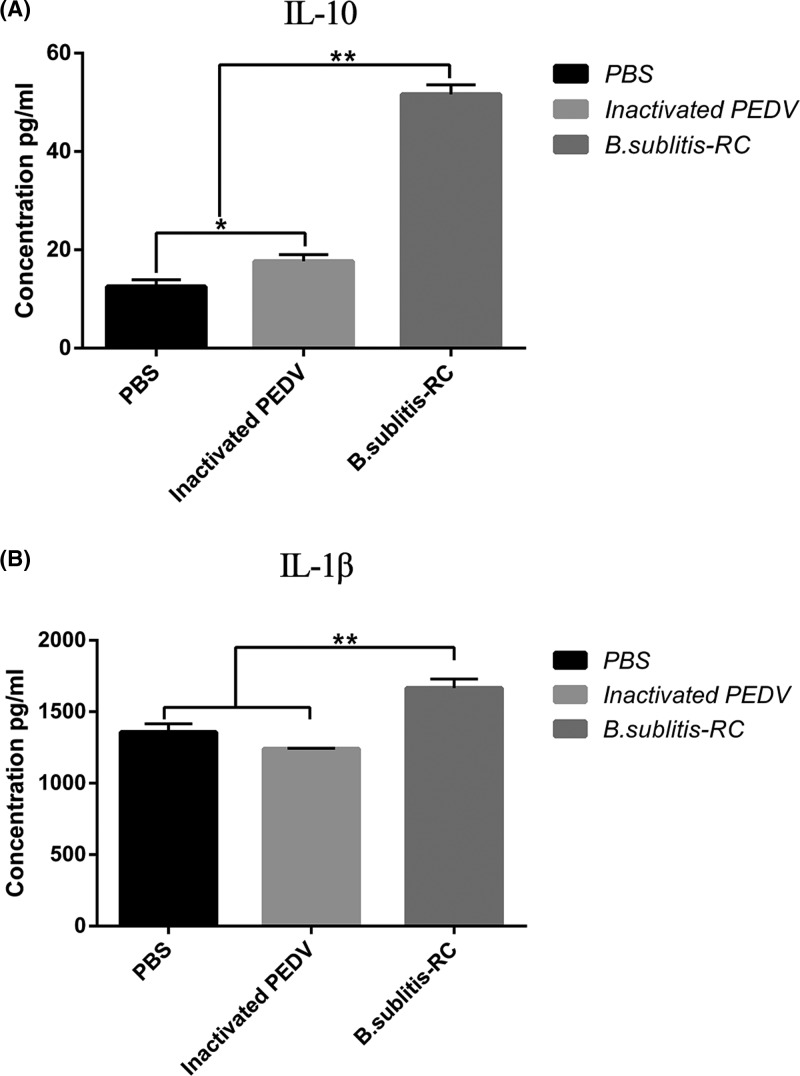
Oral administration *B. subtilis*-RC regulated cytokine responses (**A**,**B**) Cytokines IL-10 (A) and IL-1β (B) were measured by ELISA. Data were shown as the mean ± S.D. *P*-value: *P<0.05, **P<0.01.

Cytokine IL-1β and IL-10 levels were determined to evaluate cellular immune responses induced by *B. subtilis*-RC as shown in Figure 6A,B. As we can see from the diagram, significantly (*P*<0.01) higher IL-1β and IL-10 were produced after oral administration with *B. subtilis*-RC than the other two groups. These all revealed that *B. subtilis*-RC could stimulate cytokines release to mediate communication with and between cells of the immune system, improving the mucosal immune response to PEDV infection.

### Oral immunization with *B. subtilis*-RC increased the titers of PEDV neutralizing antibodies

**Figure 7:**
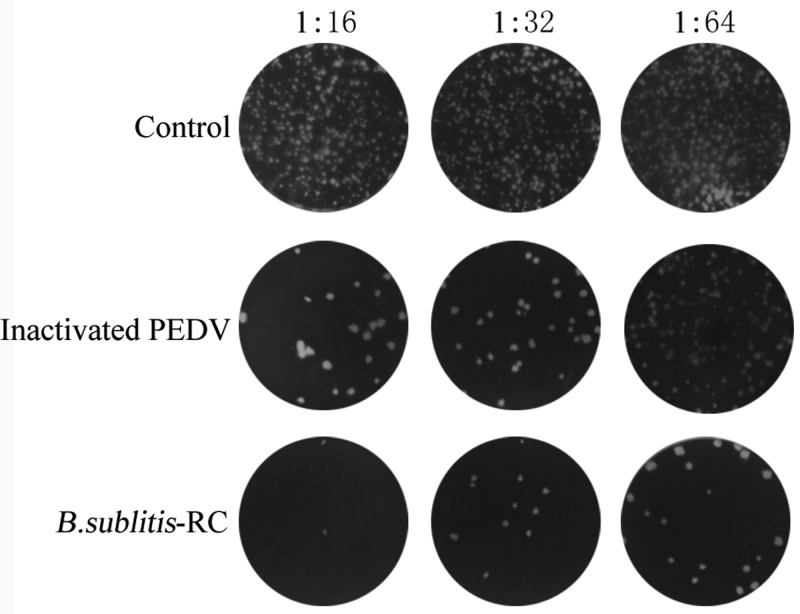
*B. subtilis*-RC induced the production of the PEDV neutralizing antibodies PEDV neutralizing antibodies were measured in intestine washing liquid by PRNT. Data were shown as the mean ± S.D. *P*-value: *P<0.05, **P<0.01.

The PEDV neutralizing antibodies were detected by PRNT assay. Oral administration with *B. subtilis*-RC could effectively reduce the plaque-forming ability of PEDV (*P*<0.01) compared with other two groups in Figure 7. This revealed that *B. subtilis*-RC could stimulate high level of PEDV neutralizing antibodies against PEDV infection.

## Discussion

Amidst the PEDV outbreak, various vaccines have been developed to control diseases and the effects are unsatisfactory. Oral vaccines can induce more robust mucosal immunity than injectable counterparts [32]. Therefore, oral immunization has appeared as an effective strategy for controlling PEDV outbreak [33].

It is now clear that effective mucosal immune response requires serum IgG and mucosal SIgA [34]. SIgA is the basis of the mucosal immune system, playing an important role in maintaining the immune homeostasis, and neutralizing the invasive pathogens. Serum IgG represents systemic immune responses. During PEDV infections, oral immunization elicits not only mucosal but also systemic immune responses very well [35]. Our data showed a strong and long-lasting anti-PEDV IgG response were detected by oral administration with *B. subtilis*-RC in piglets. Although as time went on, the antibody titers declined a little, it still stayed on overhead compared with control groups and with accordance to the changeable tendency of antibodies. The change of specific IgA showed similar results in mouth and feces mucosa. All these changes had contributed to fight PEDV infection. As the extra immunity boost, *B. subtilis*-RC reduced the ability of pathogens to cross the intestinal mucosa and the systemic spread of invasive pathogens [36].

The mucosal immune system generates immune responses through immune cells that reside in mucosal compartments. T lymphocytes residing in the mucosa play important roles in mucosal immunity [37]. We further explored the species, amounts, and distribution of T lymphocytes in the intestine mucosa. CD3 is a fundamental cell surface marker of T lymphocytes [38]. The result showed that the number of CD3^+^ T lymphocytes significantly increased, and these revealed that *B. subtilis*-RC could stimulate T-cell maturation. According to the molecules expressed on the cell surface, T lymphocytes can further divide into T helper cells (CD4^+^ T cells) and cytotoxic T cells (CD8^+^ T cells) [39]. Furthermore, we observed that the ratio of CD4^+^/CD8^+^ T cells increased by oral administration. The CD4/CD8 ratio measures the ratio of T helper cells to cytotoxic T cells. Therefore, we could see that oral administration *B. subtilis*-RC could strengthen Th1 immune response by raising the ratio of CD4^+^/CD8^+^ T cells.

Small intestine morphology can directly reflect the intestinal health and plays an important role in maintaining the intestine immune system [40]. The early stage of PEDV infection is frequently accompanied by necrosis and exfoliation of infected villous epithelial cells, ultimately resulting in acute, severe villous atrophy [41]. Therefore, the effective work of maintaining intestine morphology is a good indicator for assessing the efficacy of vaccines. After oral administration with *B. subtilis*-RC, we found the area of PPs expanded significantly. PPs are small masses of lymphatic tissue and form an important part of the immune system by recruiting and inducting the T cells to prevent the growth of pathogens in the intestines. Furthermore, an increase in the number of IELs demonstrated the effectiveness of *B. subtilis*-RC. Moreover, the villi length of ileum showed some encouraging results that a well-formed intestine morphology came into being by *B. subtilis*-RC. The satisfactory intestine morphology was the first step on the road against PEDV infection. Several morphology results proved that *B. subtilis*-RC could remarkably maintain the intestine morphology and form comprehensive protection.

As previously mentioned, oral administration with *B. subtilis*-RC could stimulate T-cell proliferation and differentiation and modulate the immune response. Moreover, cytokines are small-molecule proteins with wide biological activity, synthesized and secreted by immune cells and some non-immune cells [42]. As a cell signaling molecule, it mainly acts to regulate immune responses, participating in the differentiation and development of immune cells, mediating inflammatory responses, stimulating hematopoiesis, and participating in tissue repair. Previous studies had demonstrated that PEDV inhibited both NF-κB and pro-inflammatory cytokines [43]. Therefore, cytokines are a key indicator for evaluating the ability of a vaccine to stimulate immune responses. In this study, we had observed that IL-1β and IL-10 increased (*P*<0.01) remarkably. IL-1β as one of the earliest pro-inflammatory cytokines and is centrally involved in the initiation and regulation of inflammatory and innate immune responses. Research had shown that IL-1β could significantly up-regulate the local and systemic immune tissues post microbial infection [44]. In addition, IL-10 is a potent anti-inflammatory cytokine that plays an essential role in preventing inflammatory and autoimmune pathologies [45]. In summary, both data showed that oral administration with *B. subtilis*-RC regulated and enhanced immunity by up-regulating cytokines IL-1β and IL-10.

In conclusion, the present results demonstrated that oral immunization with *B. subtilis*-RC could effectively induce local mucosal and systematic immune responses against PEDV infection, while enhancing and regulating the immune function by raising the ratio of CD4^+^/CD8^+^T cells and cytokines IL-1β and IL-10, thus pointing to a promising oral vaccine candidate for PEDV infection in piglets.

## Abbreviations

*B. subtilis*-RC: Recombinant *Bacillus subtilis*
CFU: Colony forming units
COE: Core epitope
DAB: Diaminobenzidine
DC: Dendritic cell
DPBS: Dulbecco’s PBS
GI: Gastrointestinal
HE: Hematoxylin eosin
HRP: Horseradish peroxidase
IEC: Intestinal epithelial cell
IEL: Intraepithelial lymphocyte
IHC: Immunohistochemistry
IL: Interleukin
LP: Lamina propria
mAb: Monoclonal antibody
NF-κB: Nuclear factor κB
PED: Porcine epidemic diarrhea
PEDV: PED virus
PP: Peyer’s patch
PRNT: Plaque reduction neutralization test
RPMI: Roswell park memorial institute
SABC: Strept avidin-biotin complex
SIgA: IgA^+^ secreting antibody
Th: Helper T cell
